# Factors affecting the willingness of patients with type 2 diabetes to use digital disease management applications: a cross-sectional study

**DOI:** 10.3389/fpubh.2023.1259158

**Published:** 2023-10-23

**Authors:** Mingjiao Zhang, Hao Zhang, Rong Zhu, Huiqi Yang, Mengjie Chen, Xiaoxia Wang, Zhe Li, Zhenzhen Xiong

**Affiliations:** ^1^School of Nursing, Chengdu Medical College, Chengdu, Sichuan, China; ^2^West China Second University Hospital, Sichuan University, Chengdu, Sichuan, China; ^3^The 3rd Affiliated Hospital of Chengdu Medical College, Pidu District People's Hospital, Chengdu, Sichuan, China; ^4^Nanbu Country People's Hospital, Nanchong, Sichuan, China; ^5^Mental Health Center, West China Hospital, Sichuan University, Chengdu, Sichuan, China; ^6^Sichuan Clinical Medical Research Center for Mental Disorders, Chengdu, Sichuan, China

**Keywords:** type 2 diabetes, mobile medical, TAM, intention to use, eHealth literacy theory

## Abstract

**Background:**

The global burden of type 2 diabetes has significantly increased, leading to a considerable impact on healthcare systems worldwide. While the advent of mobile healthcare has provided some relief by addressing the shortage of certain medical resources, its adoption among the Chinese population remains relatively low. To extend the benefits of mHealth to a greater number of Chinese diabetic patients, it is essential to investigate the factors that influence their willingness to utilize it and implement targeted interventions based on these influencing factors. The Technology Acceptance Model (TAM) is widely employed to examine users' ultimate usage behaviors, and previous studies have indicated the potential relevance of the Perceived Risk (PR) theory and the eHealth Literacy Theory to users' usage behaviors.

**Objective:**

Our objective was to investigate the determinants that affect the willingness of Chinese patients diagnosed with type 2 diabetes patients to utilize digital disease management applications (DDMAs).

**Methods:**

We conducted a cross-sectional study of patients with type 2 diabetes in three tertiary general hospitals in Chengdu using questionnaires designed by the investigators. Participants were sampled using a convenience sampling method. The questionnaire comprised three sections: socio-demographic profile and medical history; current awareness and willingness to use digital disease management applications; and the current level of e-health literacy. Structural equation modeling was employed to assess the impact of patient awareness of DDMAs and e-health literacy on the willingness to use such DDMAs.

**Results:**

(1) Patients' attitudes toward using DDMAs were significantly influenced by perceived ease of use (*β* = 0.380, *P* < 0.001) and perceived usefulness (*β* = 0.546, *P* < 0.001); (2) Electronic health literacy exerted a significant impact on patients' perceived usefulness (*β* = 0.115, *P* = *0.0*18) and perceived ease of use (*β* = 0.659, *P* < 0.001); (3) Patients' willingness to use was significantly influenced by perceived usefulness (*β* = 0.137, *P* < 0.001) and use attitude (*β* = 0.825, *P* < 0.001).

**Conclusions:**

The present research findings hold both theoretical and practical significance, and can serve as a guide for healthcare practitioners and researchers to gain a deeper comprehension of the acceptance of digital disease management applications (DDMAs) among type 2 diabetes patients.

## 1. Background

The prevalence of diabetes and its complications have had a profound impact on global health. Over the past years, the number of diabetes patients has skyrocketed. In 2015, the number of diabetes patients worldwide reached 415 million, far exceeding the predicted number of 340 million by 2030 (1). The International Diabetes Federation reported that in 2019, the prevalence of diabetes was 9.3%, affecting 463 million people globally, with a projected rise to 10.9%, or 700 million people, by 2045 (2). Type 2 diabetes mellitus (T2DM) is predominant in adults, while children and adolescents also tend to develop it (3). Self-management plays a critical role in diabetes control, as it not only regulates the progression of the disease but also improves patients' health status and quality of life (4, 5). Therefore, people with diabetes should acquire more knowledge and skills related to the disease and enhance their ability to self-manage it (6, 7).

In the face of a large number of patients with various illnesses, traditional medical resources have become strained, and are unable to cater to the medical needs of all patients (8). Digital disease management applications (DDMAs) belong to the category of mobile health (mHealth) and have emerged as a new solution to address the aforementioned dilemma. These apps have a wide range of features including blood glucose recording, insulin management, diet carbohydrate calculation, medication reminder, doctor consultation, diet advice, and health knowledge (9–11). Numerous studies have demonstrated that diabetes patients can manage their illness through these digital applications, which can effectively regulate their health and reduce diabetes-related laboratory indicators (12, 13). Additionally, the use of mobile medicine has the potential to reduce the cost of managing the disease for patients (14). Moreover, especially in the context of public health emergencies such as the COVID-19 pandemic, the use of mobile medicine has reduced the influx of patients to hospitals, thereby reducing the risk of infection (15).

Despite the potential benefits offered by digital disease management applications and the increasing interest from patients, their implementation in practice remains limited (16). To promote the use of digital disease management, scholars in many countries have investigated the factors that affect patients' willingness to use mobile medicine. For example, British scholars Lee et al. have explored the attitude of diabetes patients toward the use of mobile medicine and found that technical considerations, service awareness, and empowerment are the main factors that affect their use (17). Iranian scholar Rangraz Jeddi et al. have investigated the use of smartphone apps to manage diseases in patients with T2DM and found that younger participants were more interested in using such apps (18). However, due to differences in national conditions between developed and developing countries, the influencing factors for the willingness to use digital disease management applications are different. China, being a developing country, has limited evidence on the influencing factors for T2DM patients' willingness to use such applications.

TAM constitutes an information technology framework designed to elucidate user adoption and engagement with emerging technologies. The model postulates that an individual's intention and conduct pertaining to technology adoption hinges upon their perception of the technology's usefulness and ease of use (19). Perceived usefulness and perceived ease of use constitute pivotal determinants within the technology acceptance model, which indirectly shapes users' inclination to adopt through their attitudes toward usage (20). Presently, the Technology Acceptance Model finds application in numerous domains, particularly in the realms of social media (21) and the Internet (22). Evidently, this model serves as a common tool for exploring the factors that impact users' willingness to engage with Internet technologies, making it equally applicable to digital disease management applications as part of the Internet technology landscape. The perceived risk theory posits that every consumer transaction carries a certain degree of risk, implying that the dimension of perceived risk is context-dependent (23) and is now gradually being extended to health-related contexts (24). Furthermore, this theory lends itself to the examination of behavioral intentions regarding usage. Scholars following the Norman perspective define eHealth literacy as the capacity to locate, comprehend, and assess health-related information derived from electronic sources and apply this information to address physical health concerns (25). It has been postulated that eHealth literacy exerts a positive influence on the willingness to embrace mHealth (26). This theory also finds relevance in the present study, emphasizing the significance of eHealth literacy among individuals with type 2 diabetes, and investigating whether it directly impacts the intention to utilize such technologies. Hence, this study endeavors to probe the usage intentions of T2DM patients and the factors that influence digital disease management applications, grounded in the patients' viewpoint and employing the technology acceptance model (TAM), perceived risk theory (PR), and eHealth literacy theory (E-HLT).

## 2. Methods

### 2.1. Study design

The current cross-sectional investigation was carried out in 2021 in the southwest region of China, utilizing the convenience sampling technique to select participants. The study included patients with T2DM who visited outpatient and inpatient services in three hospitals located in Chengdu. Inclusion criteria: ① individuals aged 18 years and above; ② Individuals diagnosed with type 2 diabetes in a secondary or tertiary healthcare facility, following the 1999 diagnostic criteria for diabetes established by the World Health Organization (WHO); ③ individuals possessing complete cognitive and behavioral abilities; ④ individuals who exhibited clear awareness, normal thinking, and expression abilities, and were willing to participate after giving informed consent. Patients who declined to participate were excluded from the study. A convenience sampling method was employed to select diabetic patients who fulfilled the eligibility criteria for participation in the survey. Following the formula for determining sample size in multifactor analysis, the sample size should be 15–20 times the number of variables influencing the analysis (27). In this study, there were a total of 28 measured variables, thus necessitating a minimum sample size of 420 cases, calculated as 15 times the number of variables. Considering a 20% allowance for invalid or missing questionnaires, a minimum of 525 questionnaires were distributed for the purpose of this research study.

### 2.2. Questionnaire

Our questionnaire consisted of three sections. The first section collected socio-demographic information from participants, such as age, gender, education level, city of residence, and monthly income. The second section utilized the Chinese version of the E-health literacy scale (eHEALS) instrument, which includes tests of application ability, judgment ability, and decision-making ability related to network health information and services. The scale has a Cronbach coefficient of 0.913, and the factor analysis load factor is between 0.692 and 0.869. The third section asked participants to provide their cognitive attitudes and willingness to use digital disease management applications, answering questions formulated by the different instrument items represented in the Technology Acceptance Model (TAM) and Perceived Risk Theory. The questionnaire was divided into five dimensions: perceived usefulness (4 items), perceived ease of use (4 items), perceived safety (4 items), attitude toward use (5 items), and intention to use (3 items). The questionnaire had good internal consistency, with Cronbach's a coefficient >0.8 for each dimension of the willingness to use questionnaire, a KMO value of 0.941 in the validity test, and 77.63% of the total variance explained cumulatively by the five dimensional factor rotations. To increase the research validity, five local experts with extensive experience in relevant fields supported the validation of the instrument items and offered wording modifications. Furthermore, 20 individuals with type 2 diabetes (T2DM) were randomly chosen for the pre-survey, and 20 valid questionnaires were collected. The reliability of the questionnaire related to awareness and willingness to use digital disease management applications was assessed using SPSS 22.0, indicating that the internal consistency of the questionnaire was satisfactory. Specifically, the Cronbach's α coefficient for the willingness to use questionnaire was found to be 0.944. Subsequently, the written language and the questions were refined based on the feedback received from the participants. Table 1 presents the questionnaire items employed in this study. Each item was measured using a 5-point Likert scale ranging from “strongly disagree” (1) to “strongly agree” (5).

**Table 1:** Construct items of the instrument.

**Instrument items**		**Questions**
Perceived usefulness	PU1	Diabetes management APP can manage diabetes anytime and anywhere, which I think is very convenient.
	PU2	Using diabetes management APP can improve my effectiveness in diabetes management.
	PU3	Diabetes management APP can meet my needs for diabetes management.
	PU4	In conclusion, I think the use of diabetes management APP is very useful for diabetes management.
Perceived ease of use	PEOU1	I think it's easy for me to learn to use diabetes management APP.
	PEOU2	I don't think it will take me too much time to use diabetes management APP for diabetes management.
	PEOU3	I think it's easy to master diabetes management APP.
	PEOU4	In short, I think it is easy to use diabetes management APP to manage diabetes.
Perceived safety	PS1	I'm worried that using diabetes management APP will leak my personal information.
	PS2	I'm worried that my diabetes management APP password will be stolen, resulting in economic losses.
	PS3	I'm worried about the system vulnerability of the third-party platform of diabetes management APP.
	PS4	I believe that the use of diabetes management APP is reliable and safe.
Attitude toward using	ATT1	Compared with my peers, I am more willing to try diabetes management APP to manage diabetes.
	ATT2	Even if there are risks, I intend to use diabetes management APP to manage diabetes.
	ATT3	It is a wise choice to use diabetes management APP to manage diabetes.
	ATT4	I think using diabetes management APP to manage diabetes is in line with my lifestyle.
	ATT5	In general, I support the use of diabetes management APP to manage diabetes.
Intention to use	ITU1	I'm happy to try the management method of diabetes management APP.
	ITU2	I will recommend diabetes management APP to my friends and relatives.
	ITU3	I will actively use diabetes management APP to manage diabetes.

### 2.3. Data collection

The present study received approval from the Ethics Committee of the First Affiliated Hospital of Chengdu Medical College (02020CYFYIRB-BA-129-F01). Wen Juan Xing, a Chinese online survey platform, was used to conduct the study. The researcher got written informed consent from the patients and thoroughly explained the aim of the research and the questionnaire's format to them before to the survey. Patients answered the surveys in the researcher's presence to guarantee clarity and correctness. A total of 570 questionnaires were distributed, with 559 valid questionnaires recovered, resulting in an effective recovery rate of 98.07%.

### 2.4. Statistical analysis

The collected data was subjected to statistical analysis using SPSS version 22.0 and AMOS version 23.0. Firstly, demographic data were evaluated in terms of frequency and percentage, while the mean and standard deviation of each dimension of the digital disease management application cognition and willingness to use scale were calculated. AMOS 23.0 was used to create a structural equation model to study the path association between variables. The significance of the differences was evaluated using a bilateral test with a threshold of *P* < 0.05. The Bayesian Positive Free Sampling and Bootstrap methods were used to estimate the effect value and 95% confidence interval. Statistical significance was determined at a *P* < 0.05.

## 3. Results

### 3.1. Description of respondents

The sample population consisted of 62.8% males and 37.2% females, with 14.8% of respondents aged between 18 to 45 years, 46.5% between 46 to 59 years, and 38.6% over 60 years old. Among the respondents, the majority (64.4%) had attained a middle school education level or below, followed by those with a high school diploma (23.8%) and those with an undergraduate degree or higher (11.8%). Furthermore, 40.6% of respondents reported a monthly income between RMB 2,001 and 5,000, with 29.0% earning <RMB 2000, 21.6% earning between RMB 5,001 and 10,000, and 8.8% earning over RMB 10,000. Most of the respondents (82.1%) resided in cities, with 71.2% reporting a T2DM diagnosis for <10 years, and 67.6% indicating the presence of chronic complications related to diabetes. Table 2 provides a detailed summary of the respondents' socio-demographic information.

**Table 2:** Respondents' demographic information (*n* = 559).

		**Frequency**	**Percentage (%)**
Gender	Male	351	62.8
	Female	208	37.2
Age	≤ 45	83	14.8
	46–59	260	46.5
	≥60	216	38.6
Marital status	Married	510	91.2
	Unmarried	49	8.8
Place of residence	Urban	459	82.1
	Rural	100	17.9
Education level	Primary school and below	130	23.3
	Middle school diploma	230	41.1
	High school diploma	133	23.8
	Bachelor's degree and above	66	11.8
Monthly income (RMB)	Below 2,000	162	29.0
	2,001–5,000	227	40.6
	5,001–10,000	121	21.6
	Above 10,000	49	8.8
Occupation	Personnel of government and public institutions	46	8.2
	Enterprise employees	102	18.2
	farmer	79	14.1
	Self-employed businesses	130	23.3
	Retired	202	36.1
Duration of the disease	≤ 3	201	36.0
	4–10	197	35.2
	>11	161	28.8
Chronic complications of diabetes	Yes	378	67.6
	No	181	32.4
Smartphone proficiency	Very unskilled	44	7.9
	Not skilled	119	21.3
	commonly	192	34.3
	Relatively skilled	144	25.8
	Great facility	60	10.7

### 3.2. Measurement model

The composite reliability (CR) and average variance extracted (AVE) were used to measure the reliability of each component. The CR values for the constructs in this study ranged from 0.820 to 0.967, exceeding the proposed threshold value of 0.70 (28) (Table 3). Similarly, the AVE values ranged from 0.551 to 0.880, exceeding the suggested threshold of 0.50 (29) (Table 3). These findings indicate that the constructs and measurements used in this study are reliable and well-constructed.

**Table 3:** Means, reliability, and convergent validity.

**Construct**	**Items**	**Mean (SD)**	**Standardized factor loading**	**CR**	**AVE**
Perceived usefulness	PU1	3.76 (1.71)	0.837	0.896	0.683
	PU2	3.69 (1.06)	0.828		
	PU3	3.63 (1.06)	0.801		
	PU4	3.69 (1.05)	0.840		
Perceived ease of use	PEOU1	3.14 (1.27)	0.878	0.900	0.695
	PEOU2	3.36 (1.19)	0.691		
	PEOU3	3.16 (1.27)	0.869		
	PEOU4	3.14 (1.25)	0.881		
Perceived safety	PS1	2.87 (1.34)	0.946	0.967	0.880
	PS2	2.92 (1.34)	0.958		
	PS3	2.82 (1.26)	0.953		
	PS4	3.27 (0.95)	0.894		
Attitude Toward Using	ATU1	3.35 (1.30)	0.793	0.858	0.551
	ATU2	3.04 (1.26)	0.802		
	ATU3	3.47 (1.13)	0.732		
	ATU4	3.21 (1.26)	0.787		
	ATU5	3.78 (1.12)	0.573		
Intention to use	ITU1	3.49 (1.21)	0.781	0.820	0.603
	ITU2	3.36 (1.17)	0.747		
	ITU3	3.46 (1.21)	0.800		

### 3.3. Structural model

#### 3.3.1. Model fit

A structural model was developed using the maximum likelihood (ML) method to identify any explanatory relationships. The initial model included two paths that did not show a statistically significant difference in terms of the difference in intention to use: electronic health literacy and perceived safety. After considering various factors such as correction index, standardized residuals, path coefficient *p*-values, and removing insignificant paths and variables, a modified model with satisfactory fit was achieved. Whilst evaluating the goodness of fit indices, it is recommended to use more than one indicator to evaluate model fit (30). Apart from Goodness of Fit Index (GFI) which measured slight below 0.90, all fit indices fulfill the accepted values. Both Comparative Fit Index (CFI) and Normed Fit Index (NFI) were estimated at 0.959 and 0.946 and indicates good fit (31). The Root Mean Square Error of Approximation (RMSEA) measured below 0.10 and was considered a good fit (32). The fitness indices of the proposed theoretical model are presented in Table 4.

**Table 4:** Model fit.

**Fitting index**	**CMIN/DF**	**GFI**	**AGFI**	**RMSEA**	**CFI**	**NFI**
Result	3.951	0.845	0.814	0.073	0.951	0.946
Criteria	< 3 excellent; < 5 Acceptable	>0.9 excellent; >0.7 Acceptable	>0.9 excellent; >0.7 Acceptable	< 0.05 excellent; < 0.08 Acceptable	>0.9 excellent; >0.7 Acceptable	>0.9 excellent; >0.7 Acceptable

CMIN/DF, chi-square fit statistics/degree of freedom; GFI, goodness of fit index; AGFI, adjusted goodness of fit index; RMSEA, root mean square error of approximation; CFI, comparative fit index; NFI, normed fit index.

#### 3.3.2. Analysis of influencing factors

**Figure 1:**
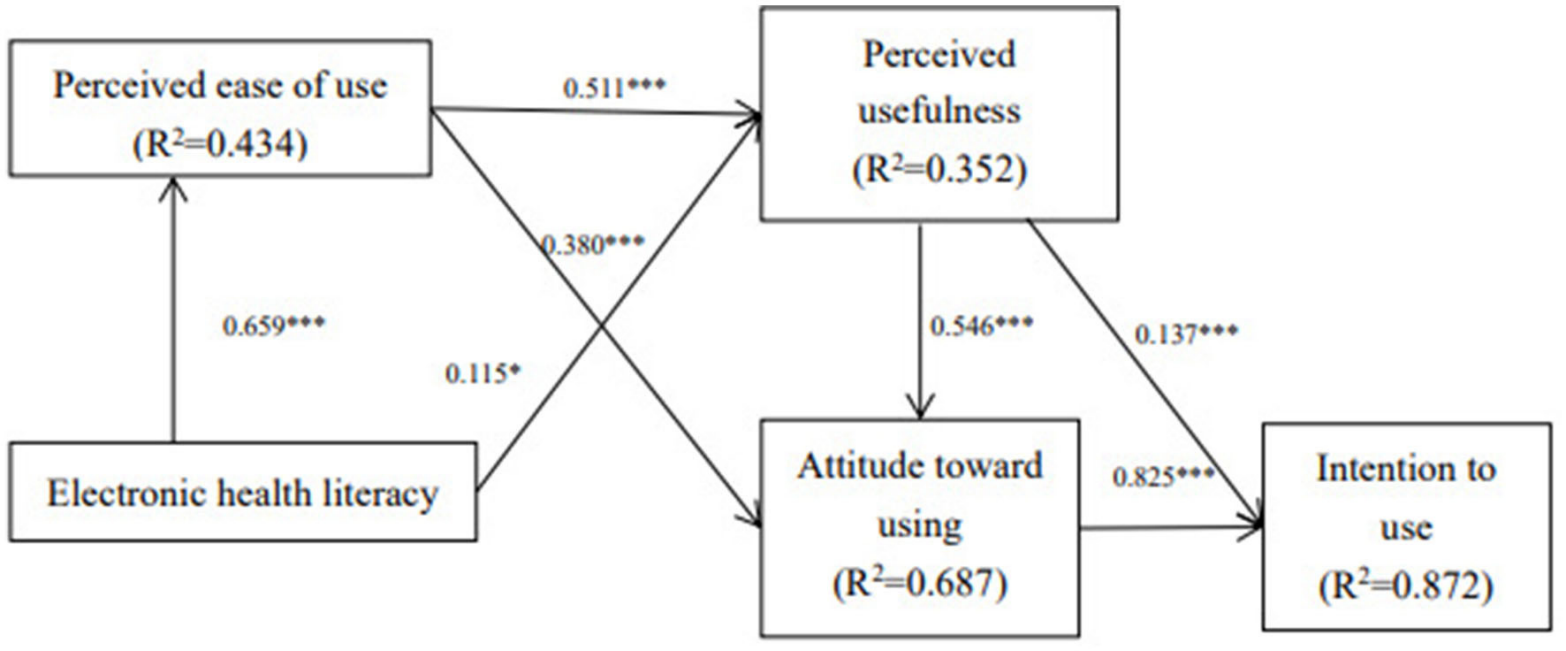
The results of the structural model (^*^p < 0.05; ^**^p < 0.01; ^***^p < 0.001).

Figure 1 and Table 5 present the results of the structural equation analysis. Perceived ease of use (*β* = 0.511, *P* < 0.001) and electronic health literacy (*β* = 0.115, *P* < 0.05) were found to predict perceived usefulness. Electronic health literacy (*β* = 0.659, *P* < 0.001) was found to predict perceived ease of use. Furthermore, perceived usefulness (*β* = 0.137, *P* < 0.001) and use attitude (*β* = 0.825, *P* < 0.001) were found to predict intention to use. In conclusion, the research model explained 43.4% of the variance in perceived ease of use, 35.2% of the variance in perceived usefulness, 68.7% of the variance in attitude toward using, and 87.2% of the variance in intention to use.

**Table 5:** Structural equation model analysis results.

**Path**	**Path coefficients**	**S.E**	**C.R**.	***P* value**
PU → ITU	0.137	0.043	3.915	< 0.001
PEOU → PU	0.511	0.039	10.250	< 0.001
PU → ATU	0.546	0.046	14.755	< 0.001
PEOU → ATU	0.380	0.034	10.986	< 0.001
ATU → ITU	0.825	0.039	20.852	< 0.001
E-heals → PU	0.115	0.035	2.374	0.018
E-heals → PEOU	0.659	0.033	18.485	< 0.001

PU, Perceived usefulness; PEOU, Perceived ease of use; ATU, Attitude toward using; ITU, Intention to use; E-heals, Electronic health literacy.

## 4. Discussion

This research emanates from China, delving into the inclination to engage with DDMAs among individuals afflicted with type 2 diabetes mellitus. The findings validate that all of the envisaged factors, barring perceived safety, exhibit robust and affirmative correlations with the proclivity to employ DDMAs among patients with type 2 diabetes mellitus. Notably, perceived usefulness and attitude toward utilization directly and positively impact the disposition to adopt, while the influence of perceived ease of use on willingness to use is mediated indirectly through perceived usefulness or attitude toward utilization. Furthermore, the impact of e-health literacy on willingness to use operates indirectly through perceived ease of use or perceived usefulness, thereby indirectly influencing the inclination to embrace these applications.

### 4.1. Willingness to use digital disease management applications

According to the study, the inclination of individuals with T2DM toward utilizing DDMAs is moderately to highly favorable, with scores exceeding 3 (out of 5) for each of the dimensions related to willingness to use, analogous to the discoveries of Mehbodniya (12) and Rangraz (18). Moreover, the study comprised 40% of the total population aged 60 years and above, who have a moderate to high intention to use DDMAs, consistent with the research conducted by Jaana M (33). This emphasis on the older population is justified by their significantly higher prevalence of T2DM compared to the general adult population, highlighting their heightened need for support from DDMAs. Additionally, this reflects the widespread presence of the internet and patients' confidence in the capability of DDMAs to facilitate effective disease management. The swift growth of mHealth in China over the years has led to reduced costs of utilization. Furthermore, China's policies have encouraged the advancement of “Internet + medicine” to ensure the availability of mobile healthcare benefits to all residents (34). MHealth has gained popular acceptance, particularly after COVID-19, making it easier for people with chronic diseases to manage their conditions and minimizing the need for hospital visits (35). Various channels have enabled patients with T2DM to become acquainted with or employ mHealth, gradually comprehending the benefits of this novel disease management model. Several avenues have facilitated the introduction and utilization of mobile health (mHealth) among patients with type 2 diabetes (T2DM), gradually imparting an understanding of the advantages associated with this innovative disease management approach. Consequently, there exists a necessity to further diminish the barriers for older individuals in accessing Internet-based healthcare services. Community and healthcare institutions should offer ample training opportunities to assist them in comprehending and mastering DDMAs (36). Simultaneously, DDMAs should be thoughtfully designed, considering the specific requirements and usability of this demographic, with a focus on maximizing ease of operation and addressing the disease management needs of this group.

### 4.2. Factors affecting willingness to use DDMAs

The Technology Acceptance Model (TAM) is a well-established and reliable research model used for forecasting user acceptance and adoption of health information technologies (21). This study is similar to previous studies applying the TAM in the context of general mobile medicine. The findings indicate that perceived ease of use and perceived usefulness are critical patient-centered factors that positively impact the willingness of patients with type 2 diabetes to use DDMAs, thereby validating the TAM model (37). These results are consistent with the findings of other studies that have explored the adoption of mHealth based on TAM, including Wang et al. (38), Nezamdoust et al. (39), and Harakeh et al. (37). Additionally, the research of Breil et al. (40) and Palos-Sanchez et al. (41). also supports the notion that perceived usefulness bears a direct impact on the inclination to adopt, while perceived ease of use can affect the willingness to use through its influence on perceived usefulness. Additionally, these two factors can synergistically interplay to shape attitudes toward utilization, consequently influencing the predisposition to adopt. Perceived usefulness refers to the subjective perception of how much patients believe DDMAs can benefit their disease management. T2DM patients are more likely to use DDMAs to manage their disease when they believe that these apps can help manage their disease (42). This underscores the necessity for DDMA developers to meticulously enhance application functionalities, aligning them with patients' requirements for effective disease management. By augmenting the user experience, the goal is to catalyze the wider adoption of DDMAs. Perceived ease of use refers to the degree to which patients find the diabetes management app effortless to use. T2DM patients are more positive about using the diabetes management app when they feel it is less difficult to operate and the setting functions are easier to understand (43). Particularly in the context of China, where individuals with type 2 diabetes are predominantly found among the middle-aged and older adult demographics (44), the formulation of disease management applications necessitates a meticulous consideration of the physiological decline associated with older adult patients. Factors like visual impairment and reduced manual dexterity should be attentively taken into account during the design process (45). These applications ought to be structured for effortless installation and operation, with an interface that emphasizes simplicity. Moreover, provisions for specialized assistance modes can also be incorporated to cater to the needs of older adult users. The path coefficient between perceived usefulness and use attitude is greater than that between perceived ease of use and use attitude. This indicates that patients attribute higher importance to the concrete advantages offered by disease management applications in comparison to the user-friendliness of these applications. Moreover, perceived usefulness directly impacts patients' willingness to use DDMAs, indicating that T2DM patients are highly concerned about the effectiveness of glucose management brought about by using DDMAs (46, 47). But the positive impact of perceived ease of use on perceived usefulness cannot be ignored. Patients may doubt the usefulness of mobile medical technology if they find it inconvenient or challenging to use, which can affect their attitude toward using it (48, 49).

This study further corroborates that electronic health literacy indirectly affects patients' willingness to use DDMAs. The findings demonstrate that electronic health literacy has a favorable impact on patients' perceived usefulness and perceived ease of use, aligning with previous research (50). Song et al. (51) posited e-health literacy as a potential variable influencing the sustained utilization of mHealth services by patients. They proposed that individuals possessing higher e-health literacy are more adept at effectively engaging with DDMAs for the purpose of managing their health conditions. Consequently, this elevated e-health literacy contributes to an augmented perception of the utility and user-friendliness of DDMAs (52). However, the path coefficient between e-health literacy and perceived ease of use is substantially greater than that of e-health literacy and perceived usefulness, signifying a more significant impact of e-health literacy on perceived ease of use. This parallels the discoveries made by Chisolm et al. (53). Enhancing users' e-health literacy to streamline their online access to requisite health information becomes notably feasible when confronted with novel web-based offerings. With the advent of the internet, patients with elevated electronic health literacy have greater access to pertinent health information and management services, thus facilitating their adoption of relevant mobile health services (54). Consequently, the electronic health literacy of patients emerges as a pivotal concern, demanding the implementation of strategies to elevate patients' proficiency in electronic health literacy. This equivalently signifies the necessity for relevant authorities to place substantial emphasis on providing technical educational support to older adult individuals grappling with diabetes. Embracing novel disease management paradigms and enhancing the efficacy and potential of disease management strategies are also imperative considerations. In contrast to prior research, this study integrates the perceived risk theory into the analysis. Surprisingly, the results show that perceived safety has no impact on patients' attitudes or willingness to use DDMAs (55, 56). Despite the complexities and potential security risks associated with online information, including online fraud and data breaches, patients still exhibit a willingness to use these digital tools (57, 58). One possible explanation for this is that older adult Chinese patients with T2DM, who make up a significant portion of the population, are less concerned with their online information exposure than younger people. During the questionnaire survey, some older adult patients did not believe that personal information leakage would impact their daily life or cause economic losses, which suggests a lack of awareness regarding information security (59). Additionally, diabetes management mHealth primarily involves recording patients' blood glucose, medication, diet, and exercise, with less involvement in their financial information. As a result, patients' perceived security is insufficient to affect their willingness to use such tools. Nonetheless, within the domain of perceived security, the entry score reveals a lower perceived security rating. This underscores that patients continue to harbor concerns regarding potential privacy breaches and the security of their personal assets arising from the utilization of DDMAs. Therefore, developers of DDMAs are compelled to intensify their focus on enhancing application security to effectively safeguard patients' personal privacy.

### 4.3. Theoretical and practical implications

In this study, a theoretical model was constructed to investigate the factors that influence the willingness of T2DM patients to use DDMAs. The results showed that e-health literacy had an impact on patients' perceived ease of use and perceived usefulness toward DDMAs. This provides new insights into improving the willingness of patients to use DDMAs. In contrast, the perceived safety may not significantly affect patients' intention to use DDMAs, suggesting that it is not the main concern for patients who decide to adopt DDMAs. However, due to the unique characteristics of the study population, future studies should also consider perceived security in their measurements.

Past scholarly inquiries within this realm have predominantly centered on evaluating the effectiveness of patients' engagement with disease management applications, resulting in fewer examinations of their inclination to embrace these tools. This is notably prominent when considering patients dealing with type 2 diabetes in the Chinese context. This study broadens the scope of the field, presenting significant and relevant insights that hold practical value for mHealth designers and educators in the field of diabetes health. By identifying the impact of e-health literacy on perceived usefulness and perceived ease of use, as well as the impact of perceived usefulness and perceived ease of use on use attitude, the findings can inform the development of personalized and user-friendly DDMAs services that cater to individual users' needs and preferences. Designers can reduce the complexity of DDMAs operation by providing guidance and prompts at each step of the process, which can be particularly helpful for older adult diabetes patients. Although the research did not find a significant impact of perceived security on user willingness to use, patients' low perceived security scores indicate that personal privacy protection should still be a consideration in DDMAs design. DDMAs providers should specify how personal information is used and ensure the safety of all personal data collected.

## 5. Limitations and future work

This study has some limitations that should be acknowledged. Primarily, the exclusivity of the survey's focus on Chinese respondents, coupled with the sole reliance on quantitative research methodologies, might have contributed to an aspect of incompleteness within the findings. Subsequent research endeavors could consider amalgamating qualitative and quantitative methodologies to offer a more comprehensive understanding of patients and yield a richer repository of information. As cultural differences may affect social norms, as well as users' perceptions and attitudes, it is crucial to confirm the research findings in other cultural contexts before generalizing them. Thus, we suggest conducting a cross-cultural comparison of the factors that influence the willingness to use DDMAs. Secondly, as the actual adoption rate of DDMAs in China is currently low, the results of this study may only be valid for predicting patients' behavior at this stage. Therefore, the population should be monitored in the future. Thirdly, the study only examined patients' willingness to use DDMAs, and further research could explore their actual usage behavior. Lastly, the evaluation of patients' smartphone proficiency rested on self-reports, potentially engendering subjectivity in the research outcomes. To enhance the precision of results, future studies could incorporate objective tools for assessing smartphone proficiency.

## 6. Conclusions

Our study delved into the impact of patient-centered factors on the patients' inclination to adopt DDMAs, and integrated the technology acceptance model (TAM), perceived risk theory, and e-health literacy to substantiate the acceptance model. Our complete model describes the variation in DDMA use willingness of 87.2%. Precisely, patient-centered factors exert diverse influences on their attitudes and behavioral propensities to utilize DDMAs. Specifically, patient-centered elements wield a diverse range of impacts on their attitudes and inclinations toward utilizing DDMAs. Among these, perceived usefulness and attitude toward utilization emerge as pivotal determinants of the propensity to adopt. For both diabetes health educators and developers of DDMAs, careful attention should be accorded to these determinants. This entails catering to patients' exigencies for disease management and perpetually refining and enhancing application functionalities. During the preliminary phases of DDMA design, a profound exploration of the type 2 diabetes patient group becomes imperative. This can be accomplished through surveys, interviews, and other modes to glean genuine patient requirements and precisely delineate the trajectory of functional development. Likewise, consulting medical professionals and pertinent guidelines is essential to elucidate the disease management focal points. Subsequent to this, functionalities can be meticulously set, guided by scientific principles and methodologies to ensure the platform's empirical soundness. Moreover, the indirect effects stemming from perceived ease of use and e-health literacy warrant substantial attention. Perceived ease of use considerably influences perceived usefulness and attitudes toward utilization. Simultaneously, e-health literacy exerts its influence on perceived ease of use. Hence, patient habits concerning DDMA usage demand consideration. Particularly for older adult type 2 diabetes patients, employing more intuitive formats such as imagery, comics, and videos can alleviate reading complexities and amplify engagement. Furthermore, our study underscores the significance of extending technological education support to type 2 diabetes patients. This holds true especially for the substantial portion of older adult or less formally educated patients. By facilitating patients' seamless integration into the trajectory of information technology advancement, they can efficaciously partake in and reap the benefits of technological development.

## Data availability statement

The original contributions presented in the study are included in the article/supplementary material, further inquiries can be directed to the corresponding authors.

## Author contributions

MZ: Formal analysis, Investigation, Methodology, Software, Validation, Writing—original draft. HZ: Investigation, Methodology, Writing—original draft. RZ: Investigation, Writing—review and editing. HY: Investigation, Data curation, Writing—original draft. MC: Investigation, Methodology, Data curation, Writing—original draft. XW: Investigation, Methodology, Data curation, Writing—original draft, Writing—review and editing. ZL: Methodology, Supervision, Validation, Data curation, Formal analysis, Writing—review and editing. ZX: Funding acquisition, Methodology, Supervision, Data curation, Formal analysis, Validation, Writing—review and editing.
